# The associations between gut microbiota and inflammatory skin diseases: a bi-directional two-sample Mendelian randomization study

**DOI:** 10.3389/fimmu.2024.1297240

**Published:** 2024-02-02

**Authors:** Yun Zhong, Fan Wang, Xin Meng, Lei Zhou

**Affiliations:** ^1^ Department of Dermatology, The Third Affiliated Hospital, Sun Yat-sen University, Guangzhou, China; ^2^ Department of Dermatology, Xiangya Hospital, Central South University, Changsha, China

**Keywords:** atopic dermatitis, gut microbiota, Mendelian randomization, psoriasis, rosacea

## Abstract

**Background:**

Accumulating evidence shows that dysregulation of intestinal flora is associated with inflammatory skin diseases, specifically atopic dermatitis (AD), psoriasis (PSO), and rosacea (ROS). However, the causality is still unclear.

**Objectives:**

To study the underlying causality between gut microbiota (GM) and AD, PSO, and ROS, a bi-directional two-sample Mendelian randomization (2SMR) analysis was conducted.

**Methods:**

Summary statistics of gut microbiota, AD, PSO, and ROS were extracted from large-scale genome-wide association studies (GWASs). In 2SMR analysis, in addition to the inverse variance weighted as the principal method for evaluating causal association, four different methods were also used. Sensitivity analysis and reverse 2SMR study were implemented to evaluate the robustness of 2SMR results or reverse causal relationship, respectively.

**Results:**

A total of 24 specific gut microbiota species related to AD, PSO, and ROS were identified by 2SMR analysis. After using the Bonferroni method for multiple testing correction, family *FamilyXIII* (ID: 1957) [OR = 1.28 (1.13, 1.45), *p* = 9.26e−05] and genus *Eubacteriumfissicatenagroup* (ID: 14373) [OR = 1.20 (1.09, 1.33), *p* = 1.65e−04] were associated with an increased risk for AD and PSO, respectively. The genus *Dialister* showed a negative association, suggesting a protective role against both atopic dermatitis and rosacea. Our reverse 2SMR analysis indicated no reverse causality between these inflammatory skin diseases and the identified gut microbiota.

**Conclusions:**

In summary, this study provided evidence for the causality between GM and inflammatory skin diseases. These findings suggested that supplementing specific bacterial taxa may be an effective therapy for AD, PSO, and ROS.

## Introduction

The incidence of several inflammatory skin diseases, including atopic dermatitis (AD), psoriasis (PSO), and rosacea (ROS), has increased dramatically over the past few decades, not only affecting the patient’s physical and mental health but also causing a huge financial burden on society (1, 2). Atopic dermatitis, also known as atopic eczema, is a chronic, recurrent, inflammatory, and pruritus skin disease associated with genetics (3). Psoriasis is a polygenic inherited dermatosis that presents as red scaly plaques, which can also affect the joints in some cases (4). Rosacea is a chronic inflammatory skin disease that occurs in the blood vessels and sebaceous units of the facial skin (5). Although the clinical characteristics and pathogenesis of AD, PSO, and ROS are different, they are generally related to genetic and external environmental factors. Consequently, it is necessary to identify the underlying pathogenesis in AD, PSO, and ROS.

The gut microbiome can establish a dynamic ecological balance between the host and the external environment. A disturbance in the gut microbiota (GM) balance can lead to the loss of multiple functions, such as the destruction of barrier function, disorders of inflammation, and immune function, resulting in the induction of diseases (6–8). Some studies have reported that changes in the abundance of gut microbiota may contribute to the aggravation of inflammatory skin diseases, while the potential mechanisms between the two are indistinct. With further research on the gut–skin axis, the alteration in the component and diversity of the GM can influence differentiation and metabolism in skin (9). In addition, gut microbiota also plays a vital role in regulating the immune response by maintaining the balance of T cells (10).

However, because there is no sufficient clinical evidence, whether there is a clear causal relationship between gut microbiota and inflammatory skin diseases remains doubtful. In general, the primary criterion for determining causality is a randomized controlled trial (RCT), which can be used to study the direct effects. In fact, an RCT is very complicated to complete and requires many participants and resources, and sometimes because of ethical issues, research on a certain factor is almost impossible. A Mendelian randomization (MR) study is one of the effective alternative methods (11). Genome-wide association studies (GWASs) perform high-throughput genomics techniques to identify variants associated with traits or diseases in specific and diverse populations, including single-nucleotide polymorphisms (SNPs) and copy number variants (CNVs), which can deepen our understanding of complex inherited traits in various diseases (12). Two-sample Mendelian randomization (2SMR) is a simple method of estimating the causal effect of exposure on outcome using GWAS summary data. In 2SMR analysis, SNPs, also called instrumental variables (IVs), are used for analyzing the causality between exposure and outcome. From a genetic perspective, SNPs are randomly assigned from parents to the next generation, which is conceptually similar to a randomized controlled trial (13). Therefore, 2SMR research can avoid the influence of reverse causality and eliminate the interference of confounding factors, making the research more reliable and credible.

In this study, the latest accessible large-scale GWAS summary statistics were utilized for 2SMR analysis to identify the potential causality between gut microbiomes and AD, PSO, and ROS, which could provide confidence for the research of inflammatory skin diseases and promote novel perspectives into the prevention and treatment of inflammatory skin diseases.

## Materials and methods

### Study design

**Figure 1:**
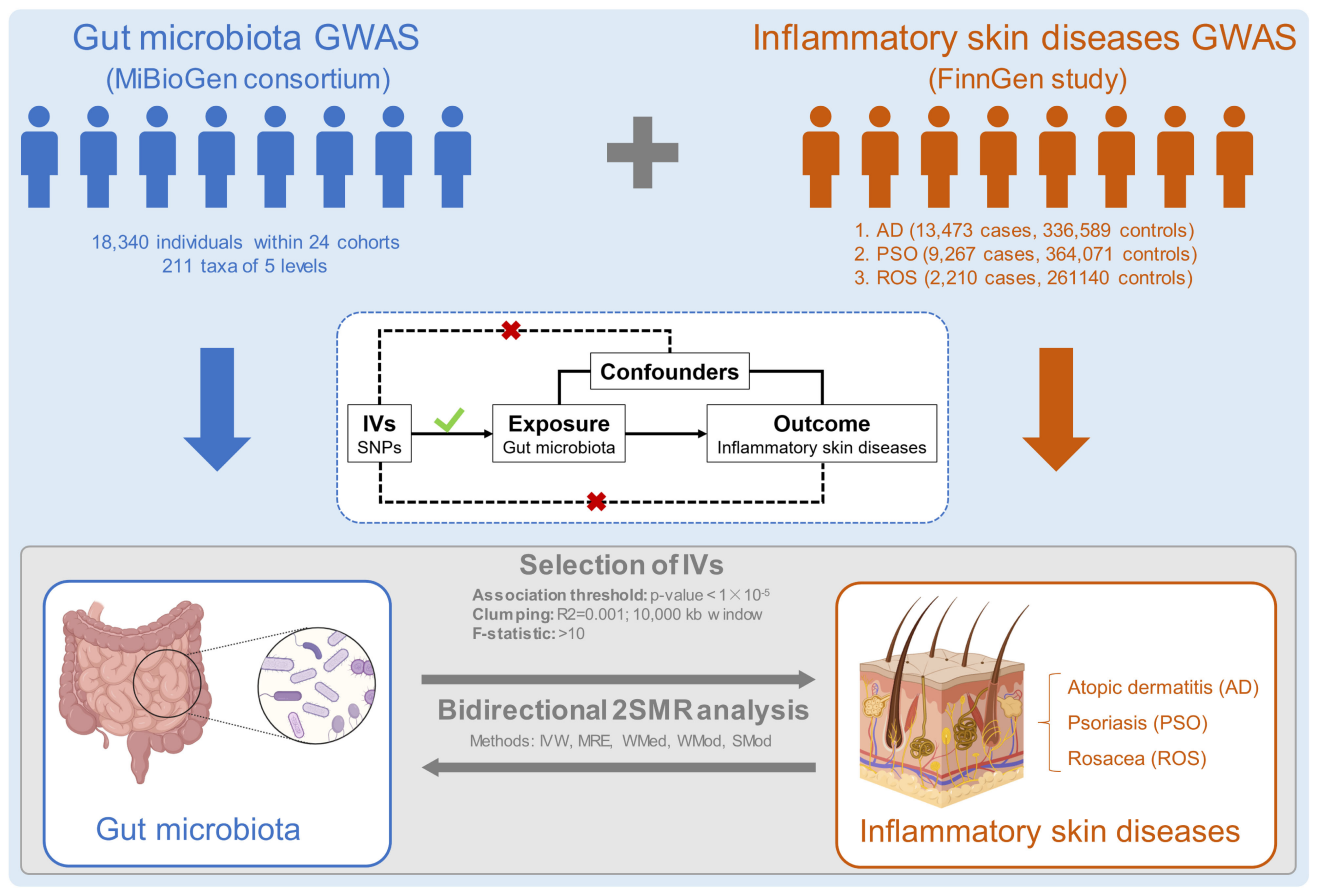
Overview of the bi-directional 2SMR study and assumptions. Summary statistics for GM and three inflammatory skin diseases were acquired from MiBioGen consortium and FinnGen study, separately. The bi-directional 2SMR study was analyzed using multiple methods, including inverse variance weighted (IVW), MR-Egger (MRE), weighted median (WMed), weighted mode (WMod), and simple mode (SMod). 2SMR, two-sample Mendelian randomization; GM, gut microbiota.

2SMR was performed to investigate the associations between gut microbiota and inflammatory skin diseases (AD, PSO, and ROS) based on GWAS summary statistics. The overall study detail is depicted in **Figure 1**. To gain convincing results, the 2SMR analysis needs to comply with three core assumptions, including strong correlation, independence, and exclusion restriction assumptions (14). The IVs used for 2SMR analysis also need to conform to the above three core assumptions, and vice versa (**Figure 1**). Reverse 2SMR analysis was used to exclude reverse causality, which can disturb causal inference. Our study is reported following the STROBE-MR guidelines (15).

### Data sources for the exposure

Genetic variants for gut microbiota were obtained from a large-scale GWAS study conducted by the MiBioGen consortium, including 18,340 individuals from 24 cohorts, most of whom are European ethnic participants (n = 13,266). For detailed clinical characteristics of all participants, please refer to the previous studies (16). This large study profiles the microbial composition with a total of 211 microbial taxa at five levels, of which 15 GM taxa without specific names were excluded.

### Data sources for the outcome

GWAS data for AD, PSO, and ROS were acquired from the latest version data by the FinnGen consortium in May 2023 (17). The diagnostic principles for AD, PSO, and ROS were based on ICD-10 standards. The GWAS statistics included 13,473 cases and 336,589 controls for AD, 9,267 cases and 364,071 controls for PSO, and 2,210 cases and 261,140 controls for ROS from a prospective cohort study involving the European population. Details of the exposures and outcomes of this 2SMR analysis can be found in **Table 1**.

**Table 1:** Details of the exposure and outcome.

Trait	Consortium	Samples	Case	Control
Exposure				
211 GM taxa	MiBioGen	18,340	/	/
Outcome				
Atopic dermatitis (AD)	FinnGen (R9)	350,062	13,473	336,589
Psoriasis (PSO)	FinnGen (R9)	373,338	9,267	364,071
Rosacea (ROS)	FinnGen (R9)	263,350	2,210	261,140

GM, gut microbiota.

### Identification of instrumental variables

To ensure the reliable causality between the gut microbiota and three inflammatory skin diseases, SNPs strongly related to GM taxa were used for IVs in our study. Considering that the quantity of available IVs at *p* < 5e−8 was quite limited, a loose cutoff of *p* < 1e−5 was set to obtain a relatively large number of IVs. In addition, genetic variations were clumped within a 10,000-kb window at the level of linkage disequilibrium (LD) and a clumping cutoff r (2) of 0.001. Then, palindromic SNPs and those not proxied were excluded from our study. Finally, weak IVs (the F-statistic of IVs < 10) were excluded from this study (18).

### Statistical analysis

In this 2SMR study, multiple methods including inverse variance weighted (IVW), MR-Egger (MRE), simple mode (SMod), weighted median (WMed), and weighted mode (WMod) were used to examine the causality between gut microbiota and AD, PSO, and ROS. If horizontal pleiotropy was not present, the IVW method can be preferred for assessing causal relationships in the 2SMR study. Meanwhile, in the presence of heterogeneity, the IVW random-effects model (IVW-RE) was performed; otherwise, the IVW fixed-effects model (IVW-FE) was used. Moreover, MRE, SMod, WMed, and WMod can be used as complementary analysis methods for IVW (19).

When the results analyzed by the 2SMR study were statistically significant (*p* < 0.05), it was suggested that there may be a causality between the gut microbiota and inflammatory skin diseases.

Moreover, we conducted a series of different analysis methods to evaluate the underlying horizontal pleiotropy, which may challenge the second 2SMR assumption. Specifically, the MR-PRESSO global test and MR-Egger intercept test can act as methods to determine whether the IVs’ horizontal pleiotropy exists. It was considered as no horizontal pleiotropy when *p* > 0.05 for both methods (20).

Additionally, we performed Cochran’s IVW Q-test to quantify the heterogeneity of IVs. We used a leave-one-out analysis as an effective method to determine potential heterogeneous SNPs. In the end, we performed a 2SMR analysis to assess whether the reverse causality of inflammatory skin diseases (AD, PSO, and ROS) and the identified GM existed. We also detected a potential reverse causal relationship by using the MR Steiger filtering test and reverse 2SMR, which is basically consistent with the 2SMR analysis above. A flowchart illustrating the process of subject selection and screening is depicted in **Supplementary Data Figure 1**. We performed all 2SMR statistical analyses using the R package of “TwoSampleMR” (version 0.5.6) (21).

## Results

### Details of instrumental variables

In summary, a total of 2,475 SNPs were identified as final IVs. According to classification criteria for microbial taxa, the above SNPs were classified into five levels: phylum, class, order, family, and genus. Specifically, the following were identified: 116 IVs in nine phyla, 214 IVs in 16 classes, 264 IVs in 20 orders, 418 IVs in 32 families, and 1,463 IVs in 119 genera. All IVs were more strongly associated with exposure than with outcome (*p*_exposure_ < *p*_outcome_). In addition, the F-statistics for each SNP were greater than 10, indicating that no IV bias existed. Details of the selected IVs are shown in **Supplementary Data Table 1**.

### Results of the 2SMR analysis

**Figure 2:**
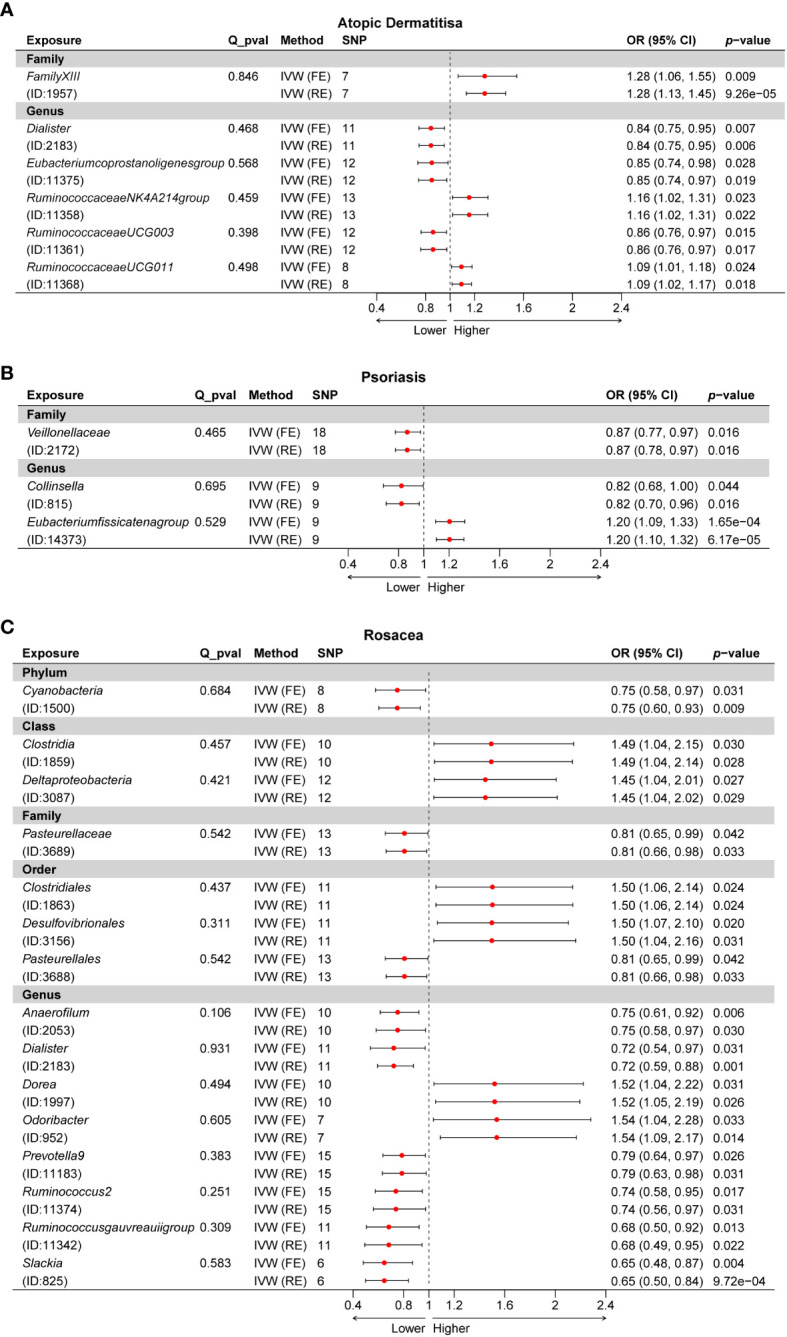
Forest plot of the causality between GM taxa with the risks of inflammatory skin diseases. **(A)** Forest plot of GM taxa associated with atopic dermatitis identified by the IVW method. **(B)** Forest plot of GM taxa associated with psoriasis identified by the IVW method. **(C)** Forest plot of GM taxa associated with rosacea identified by the IVW method. IVW, inverse-variance weighted method; GM, gut microbiota.

First, 2SMR analysis was conducted to evaluate the causal relationship between 211 GM taxa and three different inflammatory skin diseases at five different levels. The results of AD evaluated by the IVW-FE or IVW-RE suggested that the family *FamilyXIII* (ID: 1957), genus *RuminococcaceaeNK4A214group* (ID: 11358), and genus *RuminococcaceaeUCG011* (ID: 11368) were risk factors for atopic dermatitis, while the genus *Dialister* (ID: 2183), genus *Eubacteriumcoprostanoligenesgroup* (ID: 11375), and genus *RuminococcaceaeUCG003* (ID: 11361) were protective factors for atopic dermatitis (**Figure 2A**). In addition, the results of Cochran’s Q-test signified that there was no discernible heterogeneity among the selected SNPs (*p* > 0.05). Moreover, after Bonferroni correction, a significant causal relationship between the family *FamilyXIII* (ID: 1957) [OR = 1.28 (1.13, 1.45), *p* = 9.26e−05] and psoriasis remained.

Subsequently, we estimated the causal relationship between 211 GM taxa and PSO using the IVW-FE and IVW-RE methods. The results showed that genus *Eubacteriumfissicatenagroup* (ID: 14373) was associated with an increased risk for psoriasis, while family *Veillonellaceae* (ID: 2172) and genus *Collinsella* (ID: 815) were associated with a decreased risk for psoriasis (**Figure 2B**). Furthermore, the results of Cochran’s Q-test suggested the absence of heterogeneity. After Bonferroni correction, the genus *Eubacteriumfissicatenagroup* (ID: 14373) [OR = 1.20 (1.09, 1.33), *p* = 1.65e−04] remained a risk factor for psoriasis.

Third, we evaluated the causality between 211 GM taxa and ROS using the IVW-FE and IVW-RE methods. The results suggested that the class *Clostridia* (ID: 1859), class Deltaproteobacteria (ID: 3087), order Clostridiales (ID: 1863), order Desulfovibrionales (ID: 3156), genus *Dorea* (ID: 1997), and genus *Odoribacter* (ID: 952) were associated with an increased risk for rosacea, whereas the phylum Cyanobacteria (ID: 1500), family Pasteurellaceae (ID: 3689), order Pasteurellales (ID: 3688), genus *Anaerofilum* (ID: 2053), genus *Dialister* (ID: 2183), genus *Prevotella9* (ID: 11183), genus *Ruminococcus2* (ID: 11374), genus *Ruminococcusgauvreauiigroup* (ID: 11342), and genus *Slackia* (ID: 825) were protective factors for rosacea (**Figure 2C**). Similarly, the results revealed no heterogeneity in this study. Nevertheless, after Bonferroni correction, no significant effect of gut microbiota species on rosacea was observed. Interestingly, we found the genus *Dialister* was associated with a decreased risk for both atopic dermatitis and rosacea (**Supplementary Data Figure 2**).

**Figure 3:**
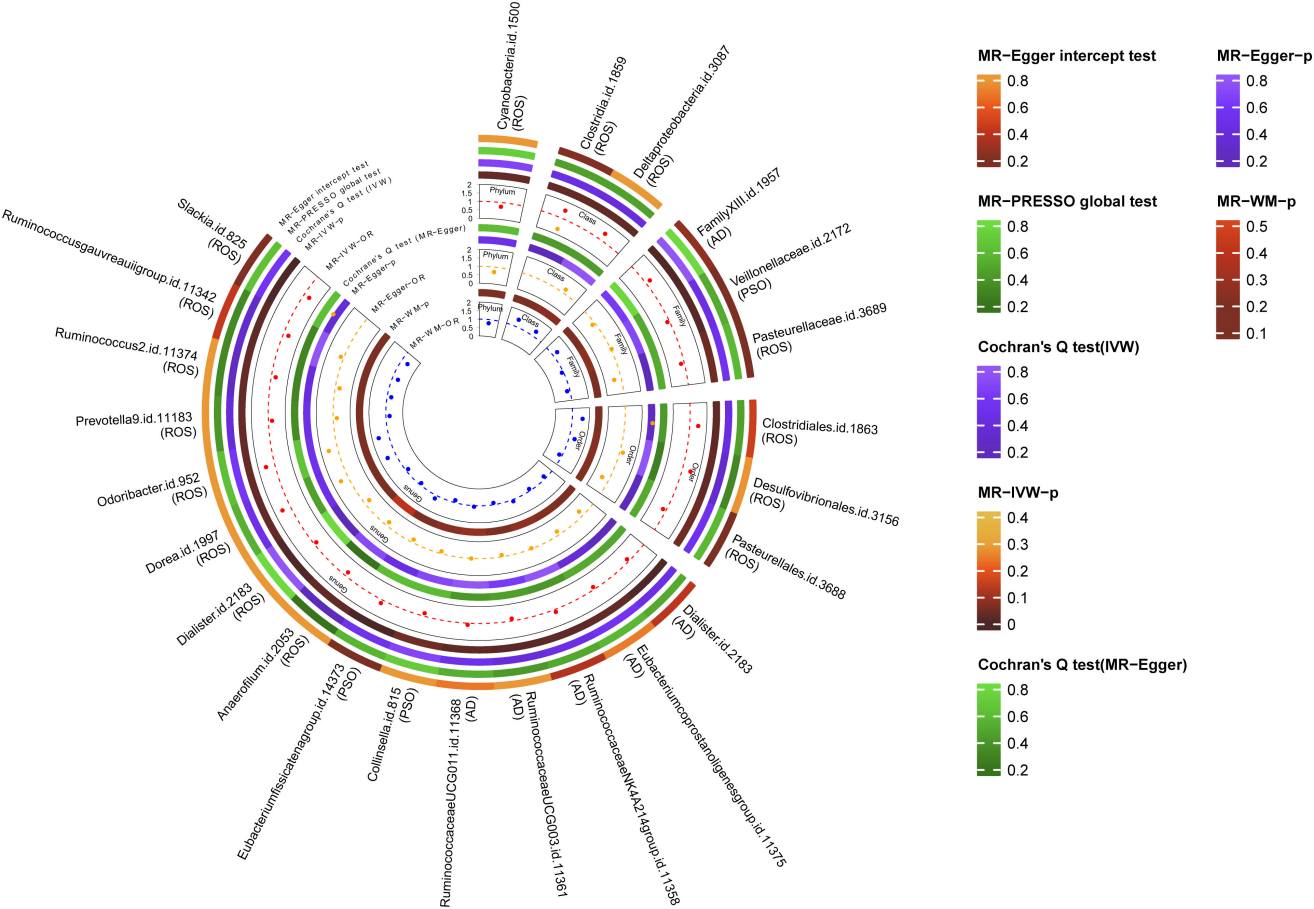
Multiple analysis of intestinal flora and inflammatory skin diseases. MR-PRESSO, Mendelian Randomization Pleiotropy Residual Sum and Outlier; IVW, inverse-variance weighted method; WM, weighted median estimator; AD, atopic dermatitis; PSO, psoriasis; ROS, rosacea.

**Figure 4:**
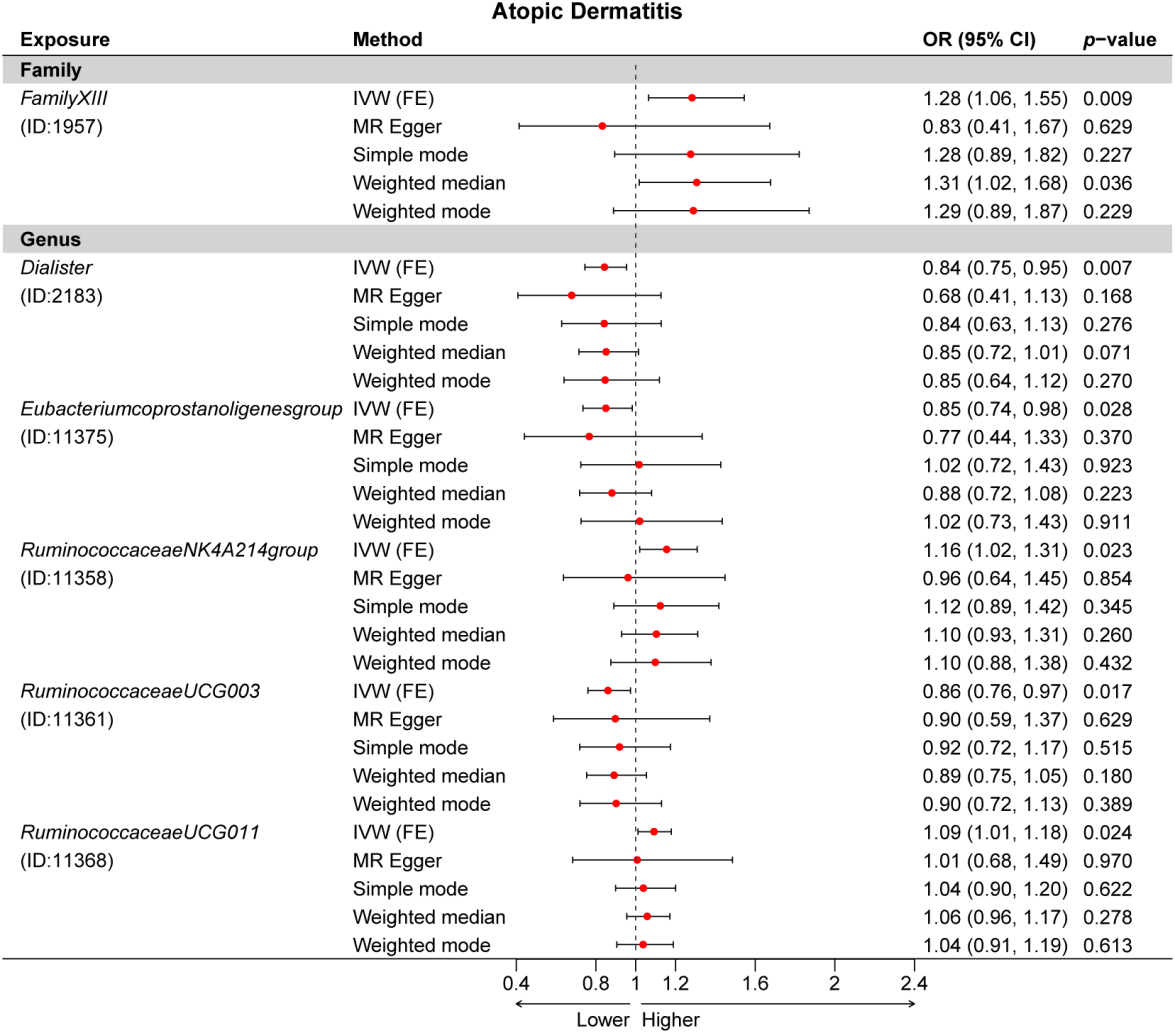
Multiple Mendelian randomization (MR) results for six GM taxa causally associated with atopic dermatitis (AD). IVW, inverse-variance weighted method; GM, gut microbiota.

**Figure 5:**
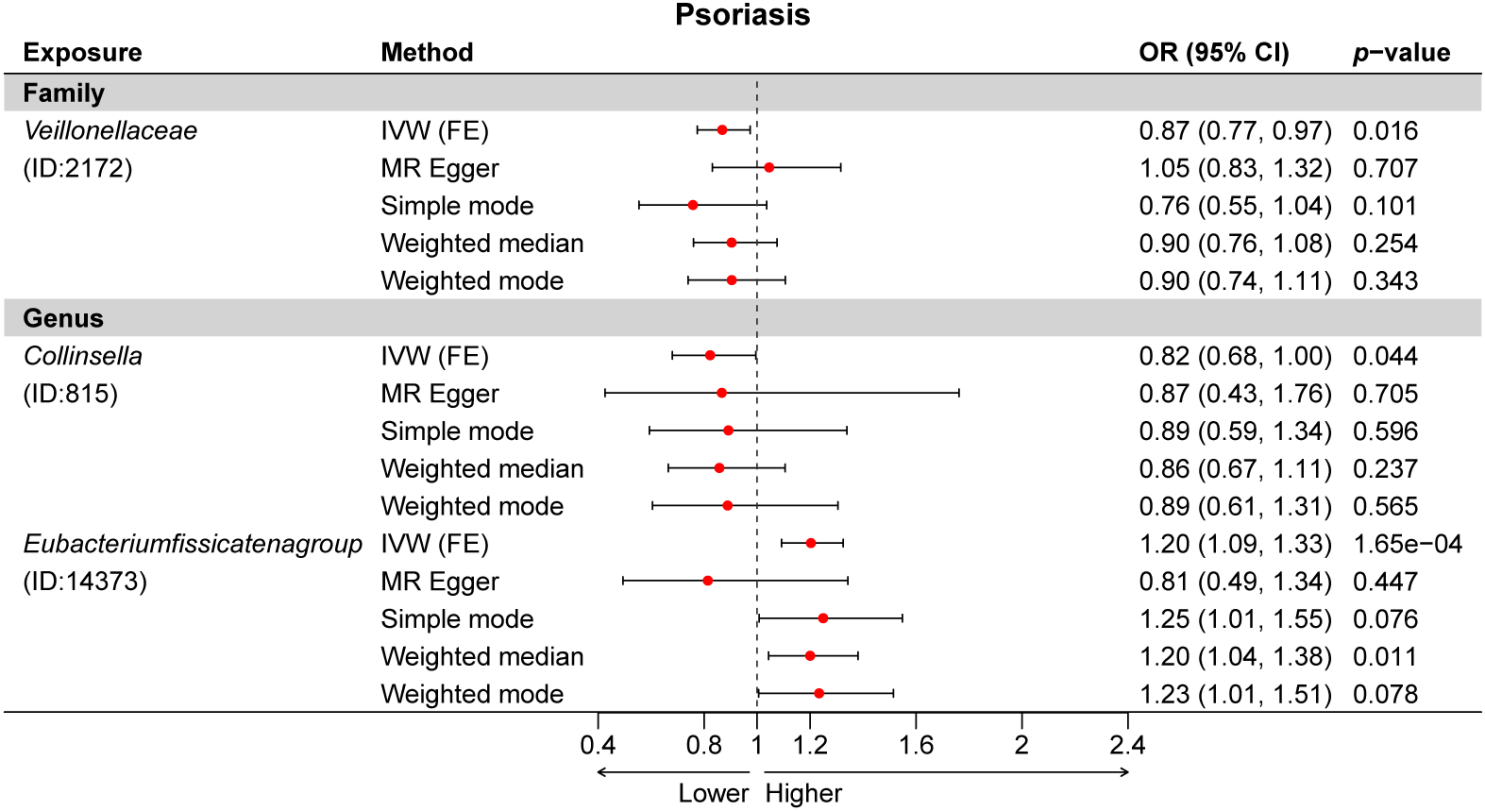
Multiple Mendelian randomization (MR) results for three GM taxa causally associated with psoriasis (PSO). IVW, inverse-variance weighted method; GM, gut microbiota.

**Figure 6:**
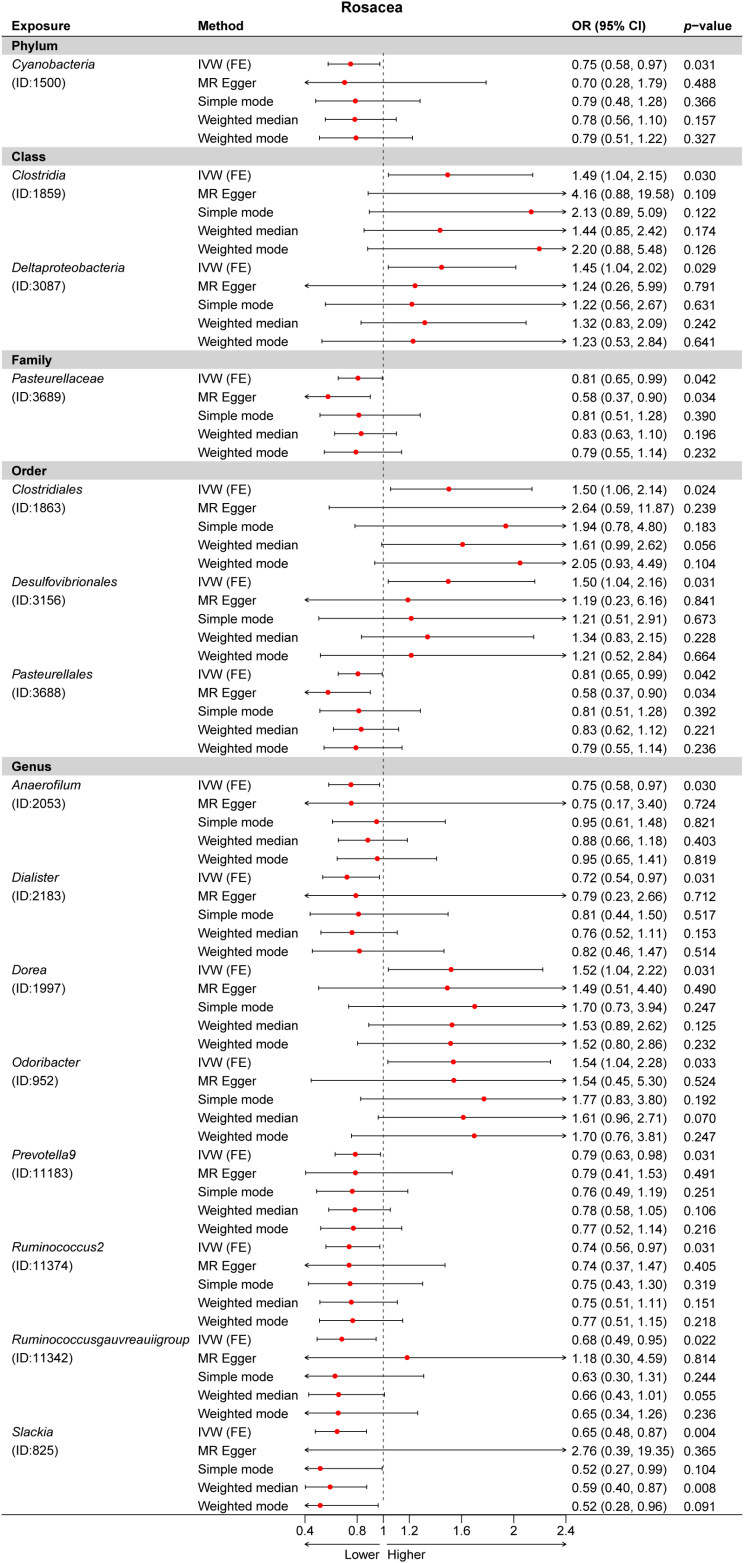
Multiple Mendelian randomization (MR) results for 15 GM taxa causally associated with rosacea (ROS). IVW, inverse-variance weighted method; GM, gut microbiota.

A total of 24 potential causal associations between gut microbiota and inflammatory skin diseases were identified using various methods (**Figure 3**). Specifically, various methods were performed, such as MR-Egger, simple mode, weighted median, and weighted mode, to evaluate the causality of these GM taxa on atopic dermatitis, psoriasis, and rosacea (**Figures 4**–**6**). As expected, the results estimated by the above methods had a similar effect as the IVW results (**Supplementary Data Figure 3**).

### Sensitivity analysis

The results of the MR-Egger intercept test indicated that there was no horizontal pleiotropy (*p* > 0.05) in IVs of six GM taxa related to atopic dermatitis, in IVs of three GM taxa related to psoriasis, and in IVs of 15 GM taxa related to rosacea. The MR-PRESSO global test showed consistent results (global *p*_MR-PRESSO_ > 0.05), indicating no horizontal pleiotropy (**Supplementary Data Table 2**). Moreover, the leave-one-out analysis suggested that the MR results were robust because the overall results were not altered by any single SNP (**Supplementary Data Figure 4**).

### 2SMR analysis after excluding confounder-related SNPs

After phenotype scanning, among the SNPs of six GM taxa related to atopic dermatitis, rs11613919, rs2523124, and rs646327 were associated with mental nervousness. Moreover, among the SNPs of three GM taxa related to psoriasis, rs12668619 and rs1442060 were associated with hyperlipidemia. Furthermore, among the SNPs of 15 GM taxa related to rosacea, rs10931481 and rs9870933 were associated with inflammation. After the above SNPs were excluded from the IVs, the causality of these GM taxa was re-assessed by the IVW method (**Table 2**). Additionally, a sensitivity analysis was conducted post-exclusion, with the leave-one-out analysis indicating robust results. This analysis showed that the overall results were not significantly altered by any single SNP, further demonstrating the stability of our findings (**Supplementary Data Figure 5**).

**Table 2:** Replicated MR analysis by the IVW method after removing confounder-related IVs.

Exposure	Outcome	*p*-Value	OR (95% CI)
Phylum			
phylum.Cyanobacteria.id.1500	Rosacea	0.031	0.749 (0.577, 0.974)
Class			
class.*Clostridia*.id.1859	Rosacea	0.03	1.493 (1.040, 2.145)
class.Deltaproteobacteria.id.3087	Rosacea	0.027	1.447 (1.042, 2.008)
Family			
family.*FamilyXIII*.id.1957	Atopic dermatitis	0.009	1.283 (1.065, 1.546)
family.*Veillonellaceae*.id.2172	Psoriasis	0.037	0.881 (0.782, 0.993)
family.Pasteurellaceae.id.3689	Rosacea	0.042	0.806 (0.654, 0.992)
Order			
order.Clostridiales.id.1863	Rosacea	0.024	1.503 (1.056, 2.138)
order.Desulfovibrionales.id.3156	Rosacea	0.02	1.498 (1.066, 2.104)
order.Pasteurellales.id.3688	Rosacea	0.042	0.806 (0.654, 0.992)
Genus			
genus.*Dialister*.id.2183	Atopic dermatitis	0.007	0.843 (0.745, 0.954)
genus.*RuminococcaceaeNK4A214group*.id.11358	Atopic dermatitis	0.023	1.156 (1.021, 1.309)
genus.*RuminococcaceaeUCG003*.id.11361	Atopic dermatitis	0.017	0.846 (0.737, 0.971)
genus.*RuminococcaceaeUCG011*.id.11368	topic dermatitis	0.024	1.092 (1.012, 1.179)
genus.*Collinsella*.id.815	Psoriasis	0.044	0.823 (0.681, 0.995)
genus.*Anaerofilum*.id.2053	Rosacea	0.006	0.752 (0.615, 0.921)
genus.*Dialister*.id.2183	Rosacea	0.031	0.721 (0.536, 0.970)
genus.*Dorea*.id.1997	Rosacea	0.031	1.520 (1.039, 2.222)
genus.*Odoribacter*.id.952	Rosacea	0.033	1.537 (1.035, 2.282)
genus.*Prevotella9*.id.11183	Rosacea	0.026	0.786 (0.635, 0.972)
genus.*Ruminococcus2*.id.11374	Rosacea	0.017	0.738 (0.575, 0.948)
genus.*Ruminococcusgauvreauiigroup*.id.11342	Rosacea	0.152	0.784 (0.563, 1.093)
genus.*Slackia*.id.825	Rosacea	0.004	0.646 (0.479, 0.871)

OR, odds ratio; CI, confidence interval; MR, Mendelian randomization; IVW, inverse variance weighted; IVs, instrumental variables.

### Reverse 2SMR analysis

We also conducted reverse 2SMR analysis and Steiger filtering between three inflammatory skin diseases and identified GM taxa. There were no significant reverse causal estimates, suggesting no causal relationships between inflammatory skin diseases and the identified GM taxa. The results of the reverse analysis are shown in **Supplementary Data Tables 3**, **4**.

## Discussion

The gut microbiome is often referred to as the “second genome” since it contains many coding genes, protecting the host from pathogen invasion, affecting metabolism, and regulating immunity (22). The intestinal flora is primarily composed of anaerobic bacteria, facultatively anaerobic bacteria, and aerobic bacteria, which establish a dynamic balance with the host and external environment, and the quantity and type remain relatively stable. Studies have shown that once this balance of intestinal flora is destroyed, it will lead to various gastrointestinal and systemic diseases (23, 24). This study determined 24 causal associations, two of which had robust causality, indicating the important role of gut microbiomes in inflammatory skin diseases, especially AD, PSO, and ROS.

Accumulating evidence has suggested that in addition to affecting the intestinal system, the gut microbiota can also influence other host organs through crosstalk, including maintaining skin homeostasis. As gut–skin axis research develops by leaps and bounds, an increasing number of studies have found that the disruption of the balance of intestinal flora will lead to the disorder of immunity and metabolism in the skin, promoting the occurrence of inflammatory skin diseases (AD, PSO, and ROS). Many relevant studies have also shown that the type and quantity of gut microbiota are negatively correlated with the onset and development of atopic dermatitis, especially in moderate and severe patients (25). Several cohort studies have suggested that levels of *Clostridium difficile*, *Escherichia coli*, and *Staphylococcus aureus* were higher among atopic dermatitis subjects compared to the healthy control (26, 27). A study in Korea showed that there are abnormal changes in the intestinal flora occurring before the onset of atopic dermatitis, such as in patients with severe atopic dermatitis with a lower quantity and diversity of propionate and butyrate bacteria in the gut (28). There has been much evidence showing the potential association between the gut microbiota and psoriasis. Several cohort studies have reported that patients with psoriasis not only have higher levels of *Prevotella* but also showed a decrease in the *Lachnospira* and *Akkermansia muciniphila* and lacked overall biological α-diversity when compared to the healthy control (29, 30). Similarly, the abundance of the gut microbiota has been noticed, and it was found that there are significant alterations between rosacea patients and healthy individuals (31). In previous studies, it has been reported that *Helicobacter pylori* infection was higher in moderate-to-severe rosacea patients compared to healthy controls, suggesting that *H. pylori* may be a risk factor for rosacea (32).

Our study identified one certain causal association between the genus *Dialister* and atopic dermatitis as well as rosacea. The genus *Dialister*, a Gram-negative, anaerobic bacterium of Firmicutes (Bacillota) (33), significantly reduced the risk of both atopic dermatitis and rosacea, which was compatible with the results of several earlier studies. As proof, according to the research report that investigated the composition and diversity of the gut microbiota in subjects with atopic dermatitis, when compared to the control group, there is a significant reduction in the genus *Dialister* (34). Our findings did not identify the causality between *Dialister* and psoriasis, while some previous studies have found that the genus *Dialister* is negatively correlated with the degree of inflammation-related markers in psoriasis patients, specifically the IL-2 receptor. The relative abundance of the genus *Dialister* can be used as an effective predictor of psoriasis activity (35). These findings suggested that the genus *Dialister* may play a vital protective and regulatory role in inflammatory skin diseases, especially in atopic dermatitis, psoriasis, and rosacea, and supplying necessary *Dialister* may contribute to the treatment or relief of symptoms.

Although the mechanisms engaged in the association among gut microbiota with inflammatory skin diseases are not thoroughly clear, some relevant evidence implies underlying pathogenesis. The abnormal activation of Th1, Th2, and Th17 cells is the most important pathogenesis of inflammatory skin diseases including atopic dermatitis, psoriasis, and rosacea, while Treg cells can negatively regulate this alteration and restore immune balance (36–38). Furthermore, several studies have discovered that the genus *Dialister* is an important producer of propionate and butyrate, which are short-chain fatty acids (SCFAs) that play a critical role in energy homeostasis and colonic activity, as well as maintaining Th1/Th2/Th17 and Treg cell balance (39, 40). In detail, the differentiation of CD4+ T cells into Treg cells can be induced by both propionate and butyrate, producing anti-inflammatory cytokine IL-10 and inhibiting the Th2-skewed and Th17-skewed inflammation. Further, it has been reported that propionate can promote the proliferation of macrophages and dendritic precursor cells, hinder the differentiation of naive T cells into Th2 cells, and thus prevent inflammation (41). Various studies have suggested that the protective effects of the genus *Dialister* against inflammatory skin diseases may be associated with SCFAs, particularly propionate and butyrate. Further RCT studies are needed to evaluate the validity and safety of probiotics as supplementary therapies for atopic dermatitis, psoriasis, and rosacea.

There are advantages to our MR study. First, we performed the first robust bi-directional 2SMR study to identify the causal associations among gut microbiota with inflammatory skin diseases (AD, PSO, and ROS), providing a novel vision into the mechanisms of the “gut–skin axis”. Second, this study not only excluded reverse causality but also eliminated the interference of confounding factors, thus ensuring the reliability of findings. Third, our finding provided 24 specific gut microbiotas associated with AD, PSO, and ROS, of which the genus *Dialister* may act as a potential probiotic in the following clinical trials and prevent or treat inflammatory skin diseases.

However, there are some limitations in our study as well, which would be noted while explaining the results. First, in addition to GWAS, other factors such as the environment can also affect diseases by altering genetics, and not all the effects of confounding factors can be eliminated by 2SMR Analysis. Second, while these identified SNPs are linked to the composition of gut microbiota, elucidating their specific connections to individual skin diseases is more challenging. This complexity partly arises from the multifactorial nature of skin diseases and the diverse roles played by gut microbiota in health and disease. Third, due to the GWAS statistics on exposures and outcomes from European ancestry, there may be overlap among the participants in this study, to a certain extent. This specificity may not fully capture the genetic diversity across different races and ethnicities, potentially leading to biased results. Additionally, the complex nature of skin diseases, influenced by various genetic, environmental, and lifestyle factors, necessitates a cautious interpretation of our findings in understanding the gut–skin axis. Fourth, the GWAS data we obtained lacked comprehensive health data for the participants. This restriction prevented us from analyzing other infections, diseases, and comorbidities that could influence our results. Future studies should aim to include such detailed health information to enhance the understanding of gut microbiota’s association with skin diseases. Finally, our findings still need to be further validated in clinical and fundamental studies. In the following MR studies, it is necessary to expand the sample size to explore the associations among gut microbiota with inflammatory skin diseases in diverse populations and at more detailed species levels.

## Data availability statement

The original contributions presented in the study are included in the article/**Supplementary Material**. Further inquiries can be directed to the corresponding author.

## Author contributions

YZ: Data curation, Visualization, Writing – original draft. FW: Writing – original draft. XM: Writing – original draft. LZ: Writing – original draft, Writing – review & editing.
