# Supplementary material for: The associations between gut microbiota and inflammatory skin diseases: a bi-directional two-sample Mendelian randomization study
Source: Front Immunol. 2024 Feb 2;15:1297240. doi: 10.3389/fimmu.2024.1297240 (PMC10869565; doi:10.3389/fimmu.2024.1297240)

**Supplementary Fig. 1 Overview of the analysis process of the causal relationship between the gut microbiome and three inflammatory skin diseases through MR analyses.**

**
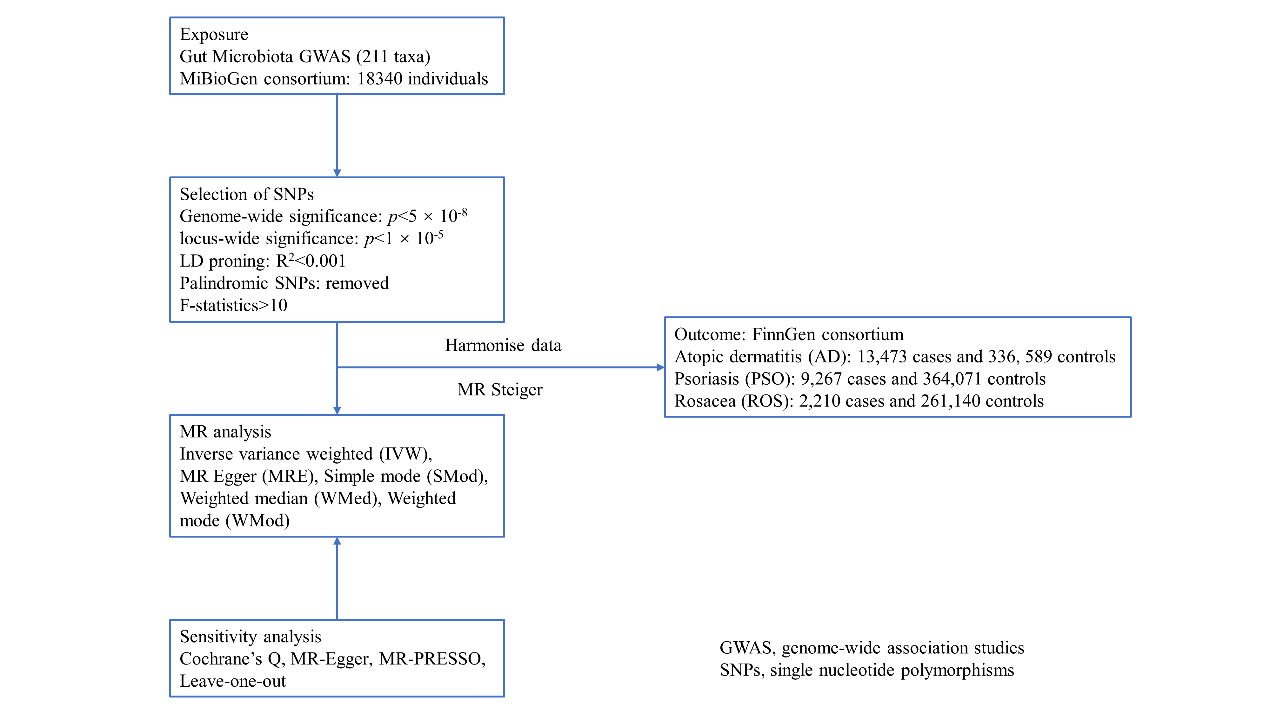
**

**Supplementary Fig. 2 Venn diagram for the identified GMs taxa causally associated with atopic dermatitis, psoriasis, and rosacea.**


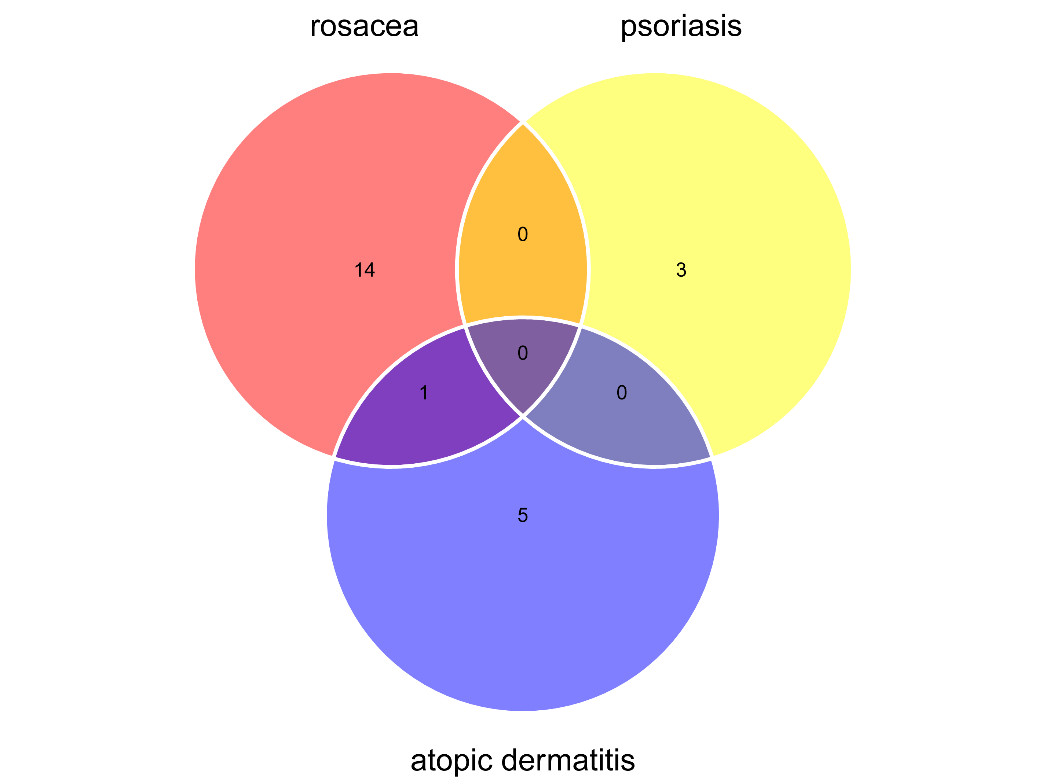


**Supplementary Fig. 3 Scatter plot visualizing MR results.** (A) for genetic correlations of 6 GM taxa with atopic dermatitis (AD) using diverse MR methods. (B) for genetic correlations of 3 GM taxa with psoriasis (PSO) using diverse MR methods. (C) for genetic correlations of 15 GM taxa with rosacea (ROS) using diverse MR methods.


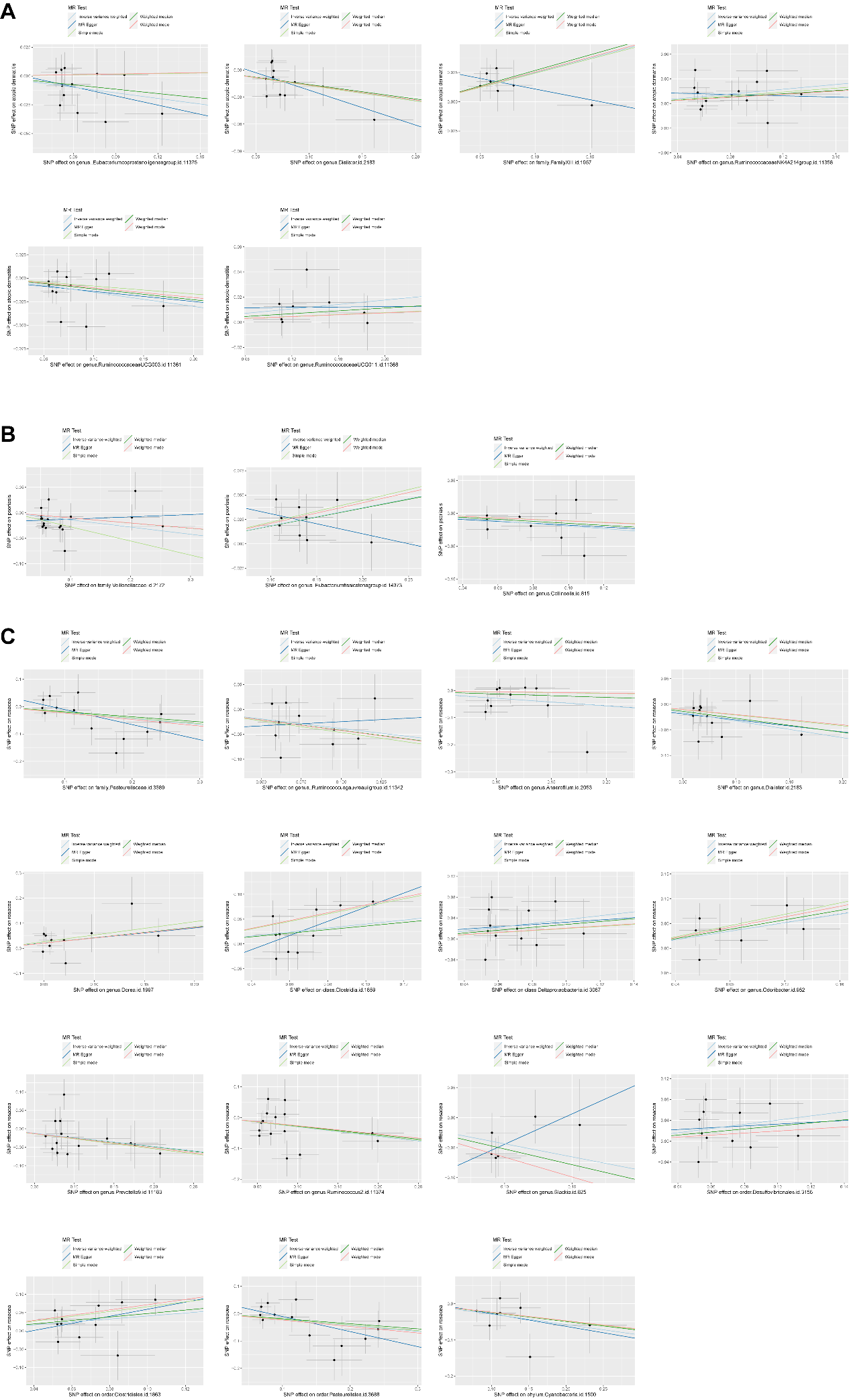


**Supplementary Fig. 4 Leave-one-out analysis.** (A) 6 GM taxa on atopic dermatitis (AD), (B) 3 GM taxa on psoriasis (PSO), and (C) 15 GM taxa on rosacea (ROS).


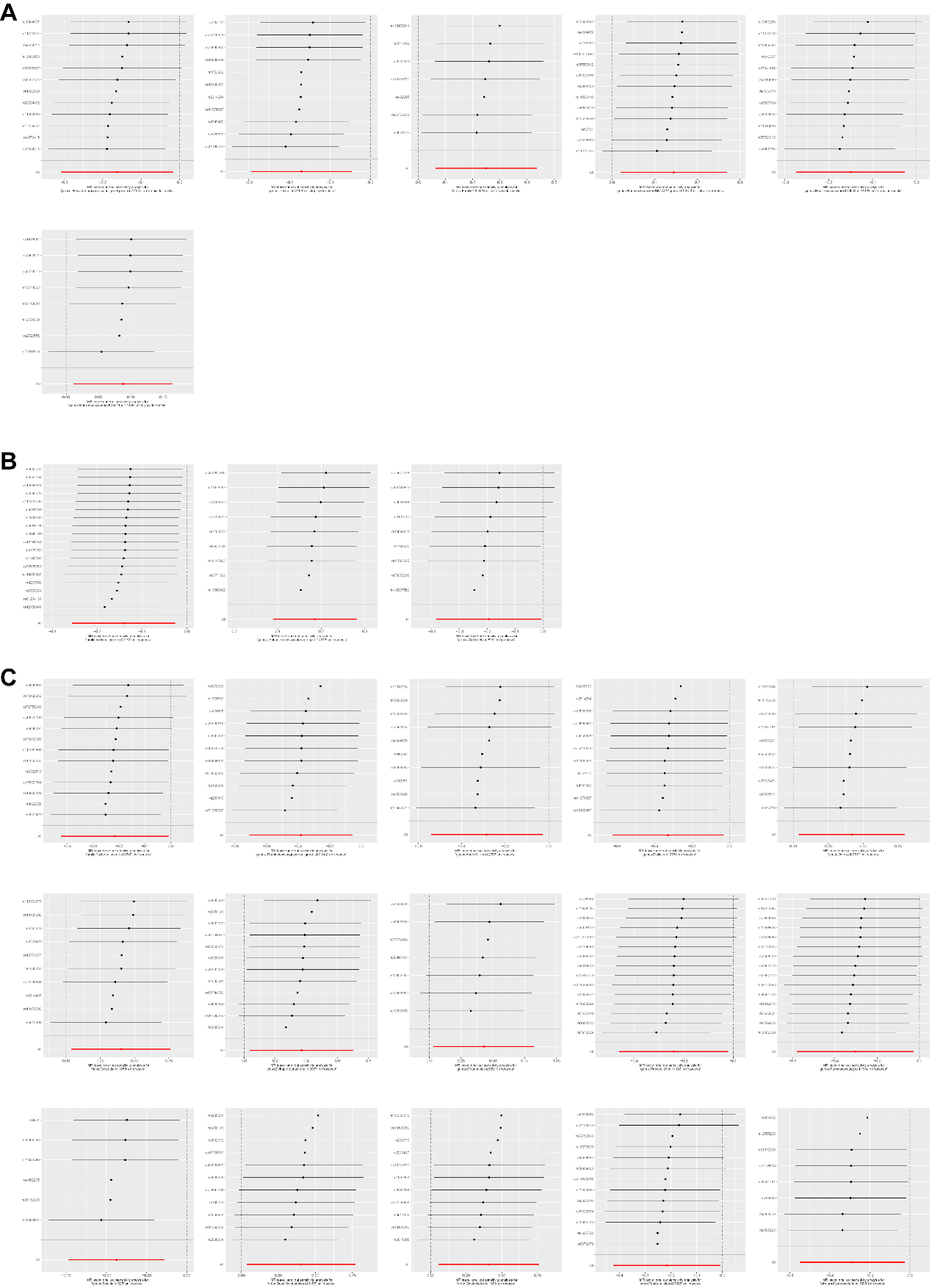


**Supplementary Fig. 5 Leave-one-out analysis after excluding SNPs linked to potential confounders.** (A) GM taxa on atopic dermatitis (AD), (B) GM taxa on psoriasis (PSO), and (C) GM taxa on rosacea (ROS).


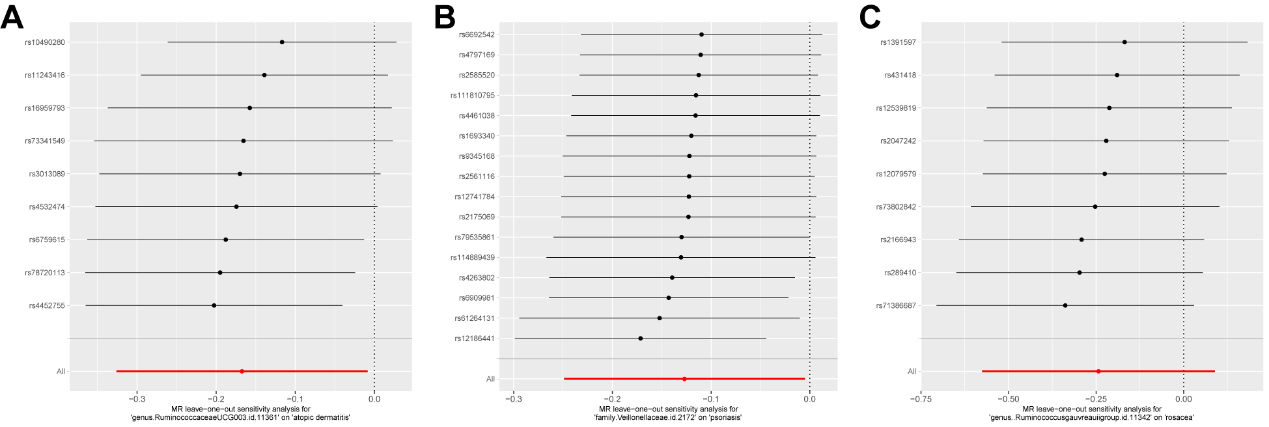

Supplement: Supplementary file 1 [file DataSheet_1.docx]
